# 

**Published:** 1878-07

**Authors:** 


JULY, 1878.

### THE AMERICAN JOURNAL OF DENTAL SCIENCE.

EDITED BY

F. J. S. GORGAS, M. D, D. D. S.,

AND

JAMES B. HODGKIN, D. D. S.

VOL. 12.—THIRD SERIES.—No. 3.

BALTIMORE:

SNOWDEN & COWMAN.

LONDON:

TRUBNER & CO., 60 PATERNOSTER ROW.

1878.

PUBLISHED MONTHLY.

$2.50 PER ANNUM.

PAYABLE IN ADVANCE.

